# Effects of the ketogenic diet on dentate gyrus and CA3 KCC2 expression in male rats with electrical amygdala kindling-induced seizures

**DOI:** 10.3389/fnins.2025.1489407

**Published:** 2025-04-08

**Authors:** Leticia Granados-Rojas, Leonardo Hernández-López, Emmanuel Leonardo Bahena-Alvarez, Tarsila Elizabeth Juárez-Zepeda, Verónica Custodio, Joyce Graciela Martínez-Galindo, Karina Jerónimo-Cruz, Miguel Tapia-Rodríguez, America Vanoye-Carlo, Pilar Duran, Carmen Rubio

**Affiliations:** ^1^Laboratorio de Biomoléculas y Salud Infantil, Instituto Nacional de Pediatría, Mexico City, Mexico; ^2^Departamento de Neurofisiología, Instituto Nacional de Neurología y Neurocirugía, Mexico City, Mexico; ^3^Laboratorio de Demencias, Instituto Nacional de Neurología y Neurocirugía, Mexico City, Mexico; ^4^Unidad de Microscopía, Instituto de Investigaciones Biomédicas, Universidad Nacional Autónoma de México, Mexico City, Mexico; ^5^Laboratorio de Oncología Experimental, Instituto Nacional de Pediatría, Mexico City, Mexico; ^6^Laboratorio de Biología Animal Experimental, Facultad de Ciencias, Universidad Nacional Autónoma de México, Mexico City, Mexico

**Keywords:** ketogenic diet, kindling, KCC2, dentate gyrus, CA3, rat

## Abstract

**Introduction:**

Ketogenic diet (KD), a high-fat, low-carbohydrate, and adequate protein diet, is a non-pharmacological treatment for refractory epilepsy. However, their mechanism of action is not fully understood. The cation-chloride cotransporter, KCC2, transports chloride out of neurons, thus contributing to the intraneuronal concentration of chloride. Modifications in KCC2 expression by KD feeding could explain the beneficial effect of this diet on epilepsy. This study aimed to determine the impact of KD on KCC2 expression in dentate gyrus layers and Cornu Ammonis 3 (CA3) strata of rats with seizures induced by amygdaloid kindling.

**Materials and methods:**

Male Sprague Dawley rats were fed a normal diet (ND) or KD from postnatal day 24 until the end of the experiment. At 6 weeks after the start of the diets, rats were subjected to an amygdala kindling epilepsy model, sham or remain intact. Glucose and β-hydroxybutyrate concentrations were quantified. The after-discharge duration (ADD), latency, and duration of stages of kindling were evaluated. In addition, KCC2 expression was evaluated using optical density. A Pearson bivariate correlation was used to determine the relationship between KCC2 expression and ADD.

**Results:**

At the end of the experiment, the KD-fed groups showed a reduction in glucose and an increase in β-hydroxybutyrate. KD reduced ADD and increased latency and duration of generalized seizures. In ND-fed animals, kindling reduced KCC2 expression in all three layers of the dentate gyrus; however, in KD-fed animals, no changes were observed. KD treatment increased KCC2 expression in the kindling group. In CA3, the pyramidal and lucidum strata showed an increase of KCC2 in KD-fed groups. Besides, the kindling had lower levels of KCC2 than the sham and intact groups. In all layers of the dentate gyrus and pyramidal and lucidum CA3 strata, the correlation indicated that the higher the KCC2 expression, the shorter the ADD during generalized seizures.

**Conclusion:**

KD reduces ADD in generalized seizures. In addition, KD has a putative neuroprotective effect by preventing the kindling-induced reduction of KCC2 expression in the molecular, granule, and hilar dentate gyrus layers and pyramidal and lucidum CA3 strata. Increased KCC2 expression levels are related to a shorter duration of generalized seizures.

## 1 Introduction

Epilepsy is a chronic disease of the central nervous system that affects individuals of all ages with a bimodal distribution. The features of this disease herald a continuous propensity to trigger abnormal, excessive, and synchronized activity in a group of brain cells with epileptic seizures as the end. This disease is considered an important public health problem worldwide (World Health Organization, 2024). According to the International League Against Epilepsy (ILAE), epilepsy is defined by any of the following conditions: (1) at least two unprovoked or reflex seizures that occur 24 or more hours apart; (2) a non-induced or reflex seizure in a person who has a 60% risk of having another seizure over the next 10 years; and (3) diagnosis of an epileptic syndrome (Fisher et al., 2014). Although, in most cases, epilepsy can be successfully treated, not all epileptic patients respond favorably to medical treatments, which can lead to drug resistant epilepsy. Drug resistant epilepsy may be defined as the failure of adequate trials of two tolerated and appropriately chosen and used antiepileptic drug schedules (whether as monotherapies or in combination) to achieve sustained seizure freedom (Kwan et al., 2010). Epilepsy and numerous neurological and neuropsychiatric disorders are known to be caused by the dysfunction of gamma-aminobutyric acid (GABA)-mediated neurotransmission (Cellot and Cherubini, 2014; Lam et al., 2023; Perucca et al., 2023; Juárez-Zepeda et al., 2024). Researchers have extensively used the amygdala electrical kindling model in the study of epilepsy neurobiology. The kindling process is the result of progressive weakening of the inhibitory system (Ryu et al., 2021; Tescarollo et al., 2023).

The strength and polarity of GABA-mediated neurotransmission are determined by the intracellular chloride (Cl^−^) ion concentration. In neurons, the Cl^−^ concentration gradient is mainly regulated by two cotransporters that belong to the cation-chloride cotransporter family; the Na^+^-K^+^-Cl^−^ cotransporter 1 (NKCC1) and the K^+^-Cl^−^ 2 (KCC2); which are encoded by Slc12a2 and Slc12a5, respectively (Kaila et al., 2014; Koumangoye et al., 2021; McMoneagle et al., 2024). The intracellular concentration of Cl^−^ is very important as it determines the postsynaptic responses to the GABA neurotransmitter, the main inhibitory neurotransmitter of the central nervous system (Belperio et al., 2022). KCC2 functions to extract intracellular Cl^−^ to maintain low levels of this ion in neurons (Duy et al., 2019). This leads to the discharge of hyperpolarizing currents regulated by GABA_A_ receptors and thus causes a reduction of epileptiform discharges or seizure activity and prompts a GABA inhibitory response to reduce neuronal excitability. In epileptic disorders, alterations in KCC2-regulated Cl^−^ transport have been identified along with a decreased efficacy of GABA_A_ receptor-mediated inhibition (Pathak et al., 2007; Lee et al., 2011; Karlócai et al., 2016; Di Cristo et al., 2018). This is attributed to the fact that an intracellular Cl^−^ accumulation leads to depolarizing currents that are regulated by GABAergic receptors and thus causes epileptiform discharges or seizure activity (Di Cristo et al., 2018). Recent studies have reported KCC2 downregulation in multiple models of epilepsy (Chen et al., 2017; Wan et al., 2018; Wan et al., 2020; Shi et al., 2023) and humans (Aronica et al., 2007; Shimizu-Okabe et al., 2011; Gharaylou et al., 2019). These studies support the importance of KCC2 regulation for neuronal intracellular Cl^−^ concentration homeostasis and proper functioning of GABA signaling (Pressey et al., 2023).

Patients suffering from refractory epilepsy (20–30% of the total) are effectively treated with various non-pharmacological treatments, such as the ketogenic diet (KD) (Martin-McGill et al., 2026; El-Shafie et al., 2023), which is characterized by a high level of fat, an adequate protein level, and strict carbohydrate restriction. KD has been introduced as a nutrition-based intervention commonly used to treat drug-resistant epilepsy since the 1920s (Wheless, 2008). KD is an adequate strategy to induce a biochemical model of fasting, where cells become less dependent on glucose energy substrate and more dependent on ketone bodies for the body’s energy needs (Fedorovich et al., 2018). To achieve this condition, the liver mitochondrial matrix has to metabolize ketone bodies. Recent research has re-established the efficacy of KD in managing epilepsy, as well as in a spectrum of neurological and neuropsychiatric disorders where a dysfunction in GABA-mediated neurotransmission is evident (Dyńka et al., 2022; Juárez-Zepeda et al., 2024).

The mechanism by which KD acts effectively in epilepsy is not clearly understood; however, the fact that KD has beneficial effects in diseases in which the KCC2 cotransporter is affected has led to the proposal that modifications in the expression of the cation-chloride cotransporter KCC2 could be at least in part the mechanism of action of this diet in epilepsy (Wang et al., 2016; Granados-Rojas et al., 2020; Murakami and Tognini, 2022). Although KD, *per se*, is known to increase KCC2 expression in the cerebral cortex (Wang et al., 2016) and dentate gyrus (Granados-Rojas et al., 2020), little is known about regional alterations of KCC2 in the hippocampus of rats fed with KD under an epilepsy model. Therefore, the present study was focused on investigating the effect of KD on the expression of the cation-chloride cotransporter KCC2, as determined by optical densitometry analysis in the dentate gyrus layers and Cornu Ammonis 3 (CA3) strata of amygdaloidal kindling seizure-induced rats.

## 2 Materials and methods

### 2.1 Animals and diets

All experimental procedures developed in the research reported in this study followed the guidelines of the Official Mexican Norm (1999) (NOM-062-ZOO-1999) and are part of project 085–2010, approved by the Research Board of the National Institute of Pediatrics. The project was also approved by the Institutional Committee for the Care and Use of Laboratory Animals (CICUAL). All efforts were made to minimize the number and suffering of the animals used in the experiments.

Male Sprague Dawley rats were bred and kept in constant controlled conditions of temperature (22°C–24°C), light:dark cycle (12:12 h, lights on from 6:00 am to 6:00 pm), and relative humidity (40%) were used. The air filter (3 microns particles) was exchanged 18 times in 1 h. At postnatal day 24, rats from 8 litters were weaned and randomly assigned to two groups as follows: normal diet-fed rats (ND) (2018S sterilized, Envigo Teklad, United States) and KD (TD 96355, Envigo Teklad, United States) (Gómez-Lira et al., 2011; Granados-Rojas et al., 2020). Both groups of animals had free access to water and their respective diets. Diets were started from weaning and were maintained throughout the experiment. Prior to the dietary treatments, the animals were subject to 8-h fasting.

### 2.2 Measurements of body weight, glucose, and β-hydroxybutyrate

The body weight, blood glucose, and β-hydroxybutyrate levels of each animal were recorded at the beginning and end of treatment. Similar to our previous study (Granados-Rojas et al., 2020), to measure blood glucose and β-hydroxybutyrate levels, a drop of blood was collected from the lateral tail tip vein and placed on glucose or β-hydroxybutyrate test strips (Abbott Laboratories) inserted into a FreeStyle Optium digital monitoring system (Abbott Laboratories glucometer) that indicated glucose or β-hydroxybutyrate concentrations (Wang et al., 2017; Doyen et al., 2024; Meeusen et al., 2024).

### 2.3 Stereotaxic surgery

After 6 weeks of ND or KD, a portion of the animals was anesthetized with ketamine (100 mg/kg intraperitoneal). Subsequently, the animals were placed in a stereotaxic device (David Kopf) to implant electrodes for stimulation and recording. The electrodes were implanted in the left basolateral nucleus of the amygdala (previous coordinates of 6.2 mm, side of 5 mm, and 1.5 mm in height). For this purpose, the interaural line was used as a reference in accordance with the Paxinos and Watson Atlas of Stereotaxy (Paxinos and Watson, 2007). Another electrode was placed in the sensory motor cortex to register the propagation of electroencephalographic activity. Each electrode was made with isolated stainless steel (0.005-inch diameter) coated with Teflon, except in the ends. A screw was implanted in the skull to serve as a source of reference. The electrodes were attached to a mini-connector and linked to the skull using dental acrylic. The skin was sutured around the mini-connector. The electrode positioning was later verified using histological staining techniques (Taddei et al., 2022).

### 2.4 Kindling model

After 10 days of postoperative recovery, the rats were placed in a silenced chamber (22.5 cm x 30 cm x 30 cm). The connector was joined to flexible cables connected to the rat with the S88 Grass stimulator and a stellate system digital polygraph. The settings of the polygraph were 50 microvolts of amplification and a filter between 3.5 and 30 Hz. The rats were stimulated daily with a 60 Hz frequency, pulses of duration of 1.0 s, and an intensity of 5 V (Goddard et al., 1969). The following parameters were measured: amygdala after-discharge duration (ADD), average ADD across 10 generalized seizures, latency or number of stimuli required to reach each kindling stage and their duration, and associated behavior according to the parameters described by Racine (1972). Stage 1: Clonus of the facial muscles, one or both eyes closed; stage 2: Oscillatory movements of the head; stage 3: Myoclonic of the forelimbs in movement; stage 4: Myoclonic movements in both extremities; stage 5: Generalized tonic–clonic seizure. Stages 1–3 were considered focal seizures, whereas stages 4 and 5 were considered generalized seizures. Sham-operated animals were implanted with electrodes but did not receive any stimulation. A part of the animal was left intact.

Finally, 6 groups of animals were used in this study:

1) IND: intact (naive) animals fed with a ND, no kindling (*n* = 8).
2) KND: animals fed with an ND with electrical amygdala kindling (*n* = 7).
3) SND: sham animals fed with a ND (*n* = 8).
4) IKD: intact (naive) animals fed with a KD, no kindling (*n* = 8).
5) KKD: animals fed with a KD with electrical amygdala kindling (*n* = 8).
6) SKD: sham animals fed with a KD (*n* = 7).

### 2.5 Tissue processing and sample collection

All animals were sacrificed 1 day after the last stimulation. At the end of treatment, rats were anesthetized with sodium pentobarbital (50 mg/kg, intraperitoneally) and transcardially perfused with 0.9% NaCl, followed by 4% paraformaldehyde in phosphate buffer, 0.1 M, pH 7.4 (PFA). The brains were then carefully removed, post-fixed in PFA overnight, and serially cryo-protected in 10, 20, and 30% sucrose at 4°C. Afterward, brain blocks containing the dorsal and ventral hippocampus of both the right and left hemispheres were sectioned in the coronal plane at 50 μm thick using a cryostat (Leica, Germany) at −21°C. Serial sections were stored in a cryoprotectant solution (25% glycerol, 25% ethylene glycol, 50% phosphate buffer 0.1 M, pH 7.4) at −20°C in 24-well plates until use. To select the sections from the serial slides per animal, a systematic random procedure consisting of choosing one of every eight sections that resulted in eight series of 12–14 sections of all rat dentate gyrus and CA3 was used. One of the series was immunohistochemically processed to immunodetection of KCC2 in each rat (Granados-Rojas et al., 2020).

### 2.6 Immunohistochemical staining

To evaluate the expression of the cation-chloride cotransporter KCC2 in the IND, KND, SND, IKD, KKD, and SKD rat groups, an immunohistochemistry protocol was carried out using a secondary biotinylated antibody according to Granados-Rojas et al. (2020). Free-floating brain tissue sections were processed in parallel at room temperature and in constant motion. Sections were initially subjected to 3 times 10-min washes with Phosphate Buffered Saline (PBS) between the change of each solution and at the end. After initial washing with PBS, sections were subjected to 1% hydrogen peroxide in PBS for 10 min. Tissues were then incubated with 20X ImmunoDNA retriever buffer (Bio SB, United States) at 65°C for 60 min, followed by incubation with the primary rabbit polyclonal antibody anti-KCC2 (1:2000; Merck Millipore, Germany, Cat. # 07–432), diluted in 5% horse serum (Gibco, United States) and 3% Triton X-100 (Merck, Germany) in PBS overnight. The antibody recognizes their total protein. The next day, the sections were washed and incubated with a secondary biotinylated goat anti-rabbit IgG antibody (1:500; Vector Laboratories, United States, Cat. # BA-1000) for 2 h, and subsequently incubated with avidin peroxidase complex (ABC kit; Vectastain; Vector Laboratories, United States, Cat. # Pk-4000) for 1 h. To determine peroxidase activity, a nickel-intensified 3,3′-diaminobenzidine (DAB; Vector Laboratories, United States, Cat. # SK-4100) solution was used for 2 ½ min. Finally, the sections were mounted on poly-L-lysine-coated slides, and entellan (Merck, Germany) was added to the slides. Then, the slides were covered with a glass coverslip. In additional sections, the primary antibody KCC2 as well as the secondary biotinylated antibody were omitted as negative controls to assess nonspecific binding. The same amount of horse serum used to replace the primary or secondary antibody resulted in a lack of any staining. Evaluation of sections was performed in a blind manner, i.e., the researcher was not aware whether the sections were from the IND, KND, SND, IKD, KKD, or SKD rat groups.

### 2.7 Optical density analysis of KCC2

The determination of KCC2 expression in the dentate gyrus and CA3 was carried out through digital densitometric analysis of acquired image color intensities. All images with identical characteristics of acquisition (objective lens, aperture condenser, light intensity, exposure time, and white balance) were taken with a MBF-CX9000 RGB CCD camera (MBF Bioscience, VT, United States) coupled to a BX-51 microscope (Olympus Corporation, Japan) and Stereoinvestigator software (MBF Bioscience, VT, United States). ImageJ software (v 1.52e, Rasband, 2018) was used to perform densitometric measurements, and the values obtained were expressed as optical density in arbitrary units. For each image, we converted RGB to 8-bit color depth, segmented the layers of interest, and measured the relative intensity of pixels in each region. The analysis was conducted at 20x in selected sections of the whole dentate gyrus and CA3. Each optical density value was normalized using background subtraction (Granados-Rojas et al., 2020). The KCC2 optical density was estimated in the molecular, granule, and hilar dentate gyrus layers, as well as in the oriens, pyramidal, lucidum, and RLM (radiatum and lacunosum-moleculare) CA3 strata. The total expression of the KCC2 cotransporter in each layer or stratum was obtained by considering the dorsal and ventral regions of both the right and left hemispheres.

### 2.8 Statistical analysis

Data on glucose, β-hydroxybutyrate, and body weight were submitted to separate three-way mixed Analyses of Variance (ANOVAs) that included the within-subject factor: Time (initial vs. final measurement) and the two between-subject factors: Diet (ND vs. KD) and Manipulation (intact, kindling, and sham).

The data from body weight as well as the KCC2 immunoreactivity (KCC2-IR) optical density in all dentate gyrus layers and all CA3 strata were subjected to separate two-way ANOVAs that included the between-subject factors: Diet (ND vs. KD) and Manipulation (intact, kindling, and sham).

For the analyses, the Mauchly’s sphericity test was performed. For all parameters with repeated measures, the Mauchly’s test indicated no sphericity (*p* < 0.05) in all cases. Therefore, the Greenhouse–Geisser correction method for sphericity was used (Verma, 2015), and the corrected results are those reported.

For all two-way ANOVAs in CA3, the Levene’s test showed equality of variances (*p* > 0.05) for all cases. On the contrary, in the dentate gyrus, Levene’s test indicated no homoscedasticity in the molecular and granule layers (*p* < 0.05), but in the hilar layer, there was equality of variances (*p* > 0.05). In all cases, the significant interactions, original degrees of freedom, and corrected probability levels are reported in addition to the partial eta squares. Tukey’s honest significant difference (HSD) tests were computed as *post hoc* analyses for the main effects found in the CA3 strata, while the different significant interactions were analyzed with the Bonferroni’s *post hoc* test.

The ADD averages were calculated for the kindling establishment and for the days corresponding to the generalized seizures. The effects of diet on these two moments were evaluated using a repeated ANOVA measures that included the between-factor Diet (ND vs. KD). *Post hoc* effects were analyzed by means of the Bonferroni’s test. The latencies (number of days needed to reach the different stages) and the duration of each Racine’s scale stage were analyzed using separate Mann–Whitney U tests.

Additionally, Pearson’s bivariate correlation test was performed to analyze whether there was a relationship between the levels of KCC2 expression in the different dentate gyrus layers and CA3 strata with the ADD generalized seizures. This test was performed independently of the group to which the animals belonged.

For all statistical analyses, the significance level was maintained at *p* ≤ 0.05. For all parameters, boxplots with individual data points were created, except for ADD. All analyses were performed using SPSS (Statistical Package for the Social Sciences), version 20.

## 3 Results

### 3.1 Body weight

**Figure 1:**
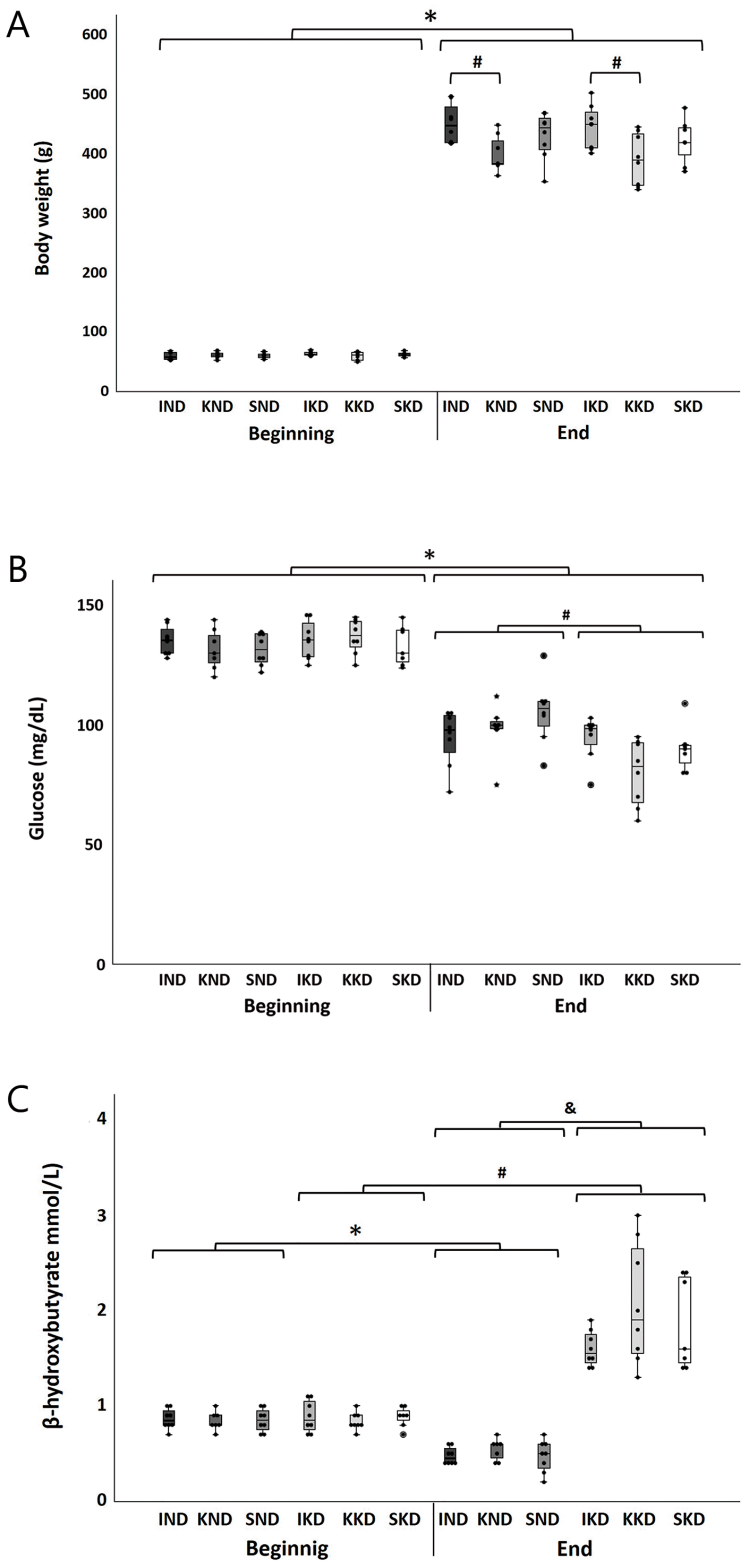
Boxplots with individual data points for each group at the beginning and end of the study of body weight **(A)**, glucose **(B)**, and β-hydroxybutyrate **(C)**. The three-way ANOVA and Bonferroni’s *post hoc* test indicated a significant body weight increase (*p* < 0.001) at the end of the experiment compared to the beginning for all groups (**A**, *). At the end, body weight was significantly lower (*p* < 0.001) for the kindling groups (KND and KKD) compared to the intact ones (IND and IKD) (**A**, #). Regarding blood glucose concentration, for all groups, there was a significant reduction (*p* < 0.001) at the end of the study regarding the beginning (**B**, *). At the end, the KD-fed groups (IKD, KKD, and SKD) presented significantly lower glucose concentrations (*p* < 0.01) than ND-fed groups (IND, KND, and SND) (**B**, #). For blood β-hydroxybutyrate concentration, at the end of the study, the ND-fed groups (IND, KND, and SND) presented a significant decrease (*p* < 0.001) regarding the beginning (**C**, *), contrary to the KD-fed groups (IKD, KKD, and SKD) that presented a significant elevation (*p* < 0.001) (**C**, #). At the end, the KD-fed groups (IKD, KKD, and SKD) presented significantly higher (*p* < 0.001) β-hydroxybutyrate concentrations than ND-fed groups (IND, KND, and SND) (**C**, &). ND, normal diet; KD, ketogenic diet; IND, ND-fed intact; KND, ND-fed with amygdala kindling; SND, ND-fed sham; IKD, KD-fed intact; KKD, KD-fed with amygdala kindling; SKD, KD-fed sham.

KD was well tolerated during the study. Assessment of body weight was performed at the beginning and end of the study. The three-way ANOVA indicated that the interactions that included the factor Diet were not significant (*p* > 0.05); therefore, there were no significant effects of diet on body weight. However, the interaction Time × Manipulation was significant (F(2, 40) = 7.202, *p* < 0.01, η^2^ = 0.265). As expected, the Bonferroni’s *post hoc* test showed that at the beginning of the study, there were no significant differences in body weight between the three types of manipulation (*p* > 0.05 for all comparisons), indicating equality of initial conditions. However, at the end of the study, the kindling groups of both diets (KND and KKD) presented a significantly lower body weight (11.69%) than the intact groups of both diets (IND and IKD) (*p* < 0.001 for all comparisons) (Figure 1A, #). For all groups, a significant body weight gain was observed at the end of the experiment when compared with the beginning of the experiment (*p* < 0.001) (Figure 1A, *).

### 3.2 Glucose

Assessment of glucose was performed at the beginning and end of the study. The three-way ANOVA indicated that the interaction Time × Diet was significant (F(1, 40) = 11.347, *p* < 0.01, η^2^ = 0.22). The Bonferroni’s *post hoc* analysis showed that in all groups, there was a significant decrease in peripheral blood glucose concentration at the end of the experiment with respect to the beginning (*p* < 0.001) (Figure 1B, *); however, this decrease was greater (34.69%) in the KD-fed groups (IKD, KKD, and SKD) than in the ND-fed groups (IND, KND, and SND) (25.10%). As expected, at the beginning of the experiment, no significant differences were observed between groups (*p* > 0.05). On the contrary, at the end of the experiment, the glucose concentration was significantly different (*p* < 0.01). The KD-fed groups (IKD, KKD, and SKD) presented a significantly lower glucose concentration (11.26%) than the ND-fed groups (IND, KND, and SND) (*p* < 0.01) (Figure 1B, #).

### 3.3 β-Hydroxybutyrate

To assess the effectiveness of the ketogenic diet, ketone bodies were measured, particularly β-hydroxybutyrate, at the beginning and end of the study. Three-way ANOVA showed that the interaction Time × Diet was significant (F(1, 40) = 164.47, *p* < 0.001, η^2^ = 0.804). As expected, the Bonferroni’s *post hoc* test indicated that at the beginning of the study, there were no significant differences (*p* > 0.5) between groups, thus showing equal initial conditions. The concentration of β-hydroxybutyrate decreased significantly (41.53%) at the end of the experiment in the ND-fed groups (IND, KND, and SND) compared with the beginning of the experiment (*p* < 0.001) (Figure 1C, *) and increased significantly (111.49%) in the KD-fed groups (IKD, KKD, and SKD) when compared with the beginning of the experiment (*p* < 0.001) (Figure 1C, #). However, at the end of the experiment, the KD-fed groups (IKD, KKD, and SKD) presented a significantly greater β-hydroxybutyrate concentration (269.47%) than the ND-fed groups (IND, KND, and SND) (*p* < 0.001) (Figure 1C, &).

### 3.4 After-discharge duration

**Figure 2:**
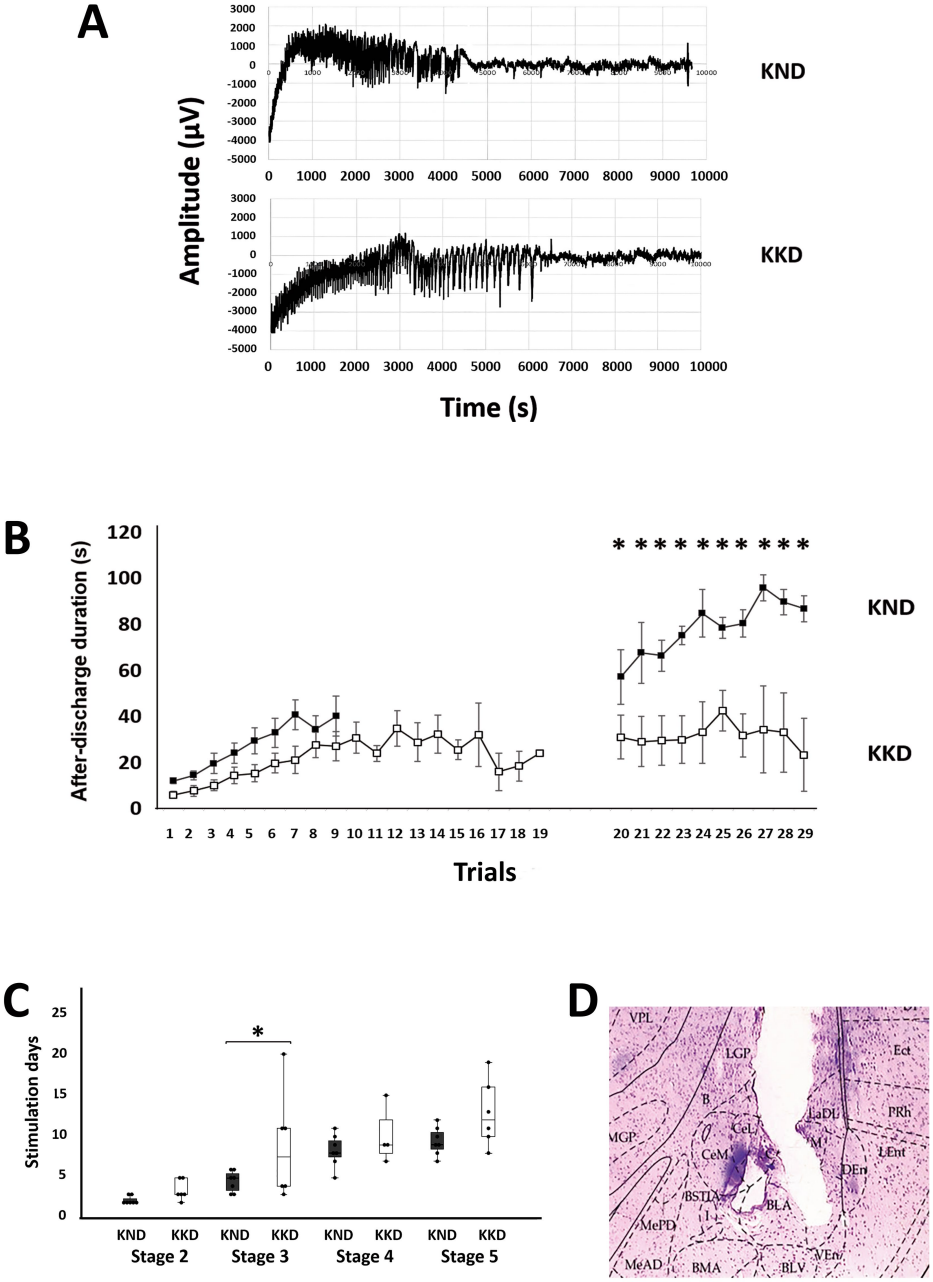
Kindling model measurements in the ND-fed group (KND) and the KD-fed group (KKD). **(A)** Representative recordings of after-discharge activity in the amygdala: at the top, the recording from the KND group; at the bottom, the recording from the KKD group. **(B)** Graphs representing the duration of after-discharge activity; values are shown as means ± standard error of the mean. Statistical significance in after-discharge was assessed using a repeated measures ANOVA, followed by Bonferroni’s test. On the left, the duration during the first 20 days. On the right, the duration of generalized epileptic activity, with evident differences in the last 10 stimuli (*F* = 16.187, *p < 0.01). Black squares represent the KND group, and white squares represent the KKD group. **(C)** Number of stimuli required to reach each Racine stage. Values are shown as boxplots with individual data points for each group. Analyses using Mann–Whitney U tests showed that the latency to reach stage 3 was significantly higher (U = 8.5, *p* < 0.05) for the KKD group than for the KND. **(D)** Microphotograph showing the placement of the electrode in the basolateral nucleus of the amygdala, using a Hematoxylin and Eosin staining technique, 40x. VPL, ventral posterolateral thalamic nucleus; LGP, lateral globus pallidus; Ect, entorhinal cortex; PRh, perirhinal cortex; LEnt, lateral entorhinal cortex; DEn, dorsal endopiriform nucleus; VEn, ventral endopiriform nucleus; LaDL, lateral amygdaloid nucleus, dorsolateral part; BLV, basolateral amygdaloid nucleus, ventral part; BLA, basolateral amygdaloid nucleus, anterior part; CeL, central amygdaloid nucleus, lateral division; B, basal nucleus (Meynert); CeM, central amygdaloid nucleus; medial division; BMA, basomedial amygdaloid nucleus, anterior part; MGP, medial globus pallidus.

Kindling model results are shown in Figures 2A-D. The repeated measures ANOVA for the kindling establishment and generalized seizures was significant (F(1, 11) = 16.187, *p* < 0.01, η^2^ = 0.595). The Bonferroni’s *post hoc* test revealed that the KD-fed kindling group (KKD) had a significantly lower ADD than the ND-fed kindling group (KND) (*p* < 0.01) only during generalized seizures (Figure 2B).

### 3.5 Latency

Analyses using Mann–Whitney U tests showed that latency to reach stage 3 was significantly higher (U = 8.5, *p* < 0.05) in the KD-fed kindling group (KKD) than in the ND-fed kindling group (KND) (Figure 2C, *). The latencies of stages 2 and 3 collapsed because they corresponded to the focal seizure conditions. Similarly, latencies of stages 4 and 5 collapsed under generalized seizure conditions. Mann–Whitney U tests indicated no significant differences in focal seizure conditions (*p* > 0.05) but did indicate the existence of significant differences in generalized seizure (*p* = 0.05), with the latency being longer in the KD-fed kindling group (KKD).

### 3.6 Stage duration

The Mann–Whitney U tests indicated that the duration of stage 4 was significantly longer (U = 1, *p* < 0.01) for the KD-fed kindling group (KKD) than for the ND-fed kindling group (KND).

### 3.7 KCC2-IR optical density

**Figure 3:**
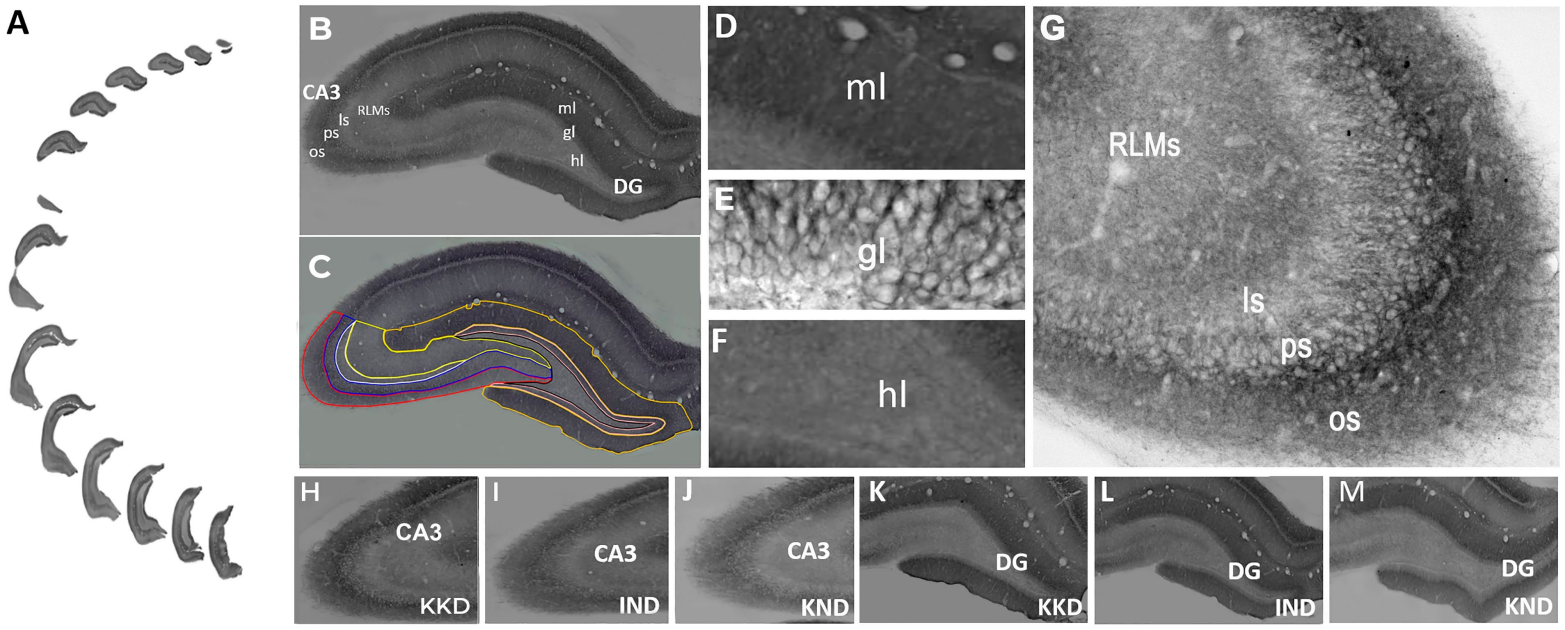
KCC2 expression in dentate gyrus and CA3. **(A)** Serial sections of the whole dentate gyrus and CA3 of the right hemisphere. **(B)** Panoramic view of molecular (ml), granule (gl), and hilar (hl) dentate gyrus layers and oriens (os), pyramidal (ps), lucidum (ls), and radiatum and lacunosum-moleculare (RLMs) CA3 strata. **(C)** The regions of interest were delimited with lines of different colors to obtain their optical density. Dentate gyrus layers: molecular (yellow line), granular (pink line), and hilar (black line). CA3 strata: oriens (red line), pyramidal (blue line), lucidum (white line), and RLM (bright yellow line). Higher magnification view of molecular **(D)**, granule **(E)**, and hilar **(F)**, dentate gyrus and oriens, pyramidal, lucidum, and RLM CA3 strata **(G)**. KCC2 immunoreactivity was observed in the plasmalemmal region (perisomal) in the granule cell body in the dentate gyrus **(E)** and in pyramidal cell CA3 **(G)**. CA3 of KD-fed with kindling rat (KKD) **(H)**, ND-fed intact rat (IND) **(I)**, and ND-fed with kindling rat (KND) **(J)**. Dentate gyrus of KKD rat **(K)**, IND rat **(L)**, and KND rat **(M)**. The KCC2 immunoreactivity was lower in the KND group compared to the IND and KKD groups. A-C, H-M, 20x. D-G, 40x.

Evaluation of the cation-chloride cotransport KCC2 expression by optical density was carried out in 12–14 sections of each rat (Figure 3A). The cytoarquitecture and diffuse staining patterns of the dentate gyrus (Figures 3B-F,K-M) and CA3 (Figures 3B-C,G-J) remained unchanged in all groups. The lamination of the dentate gyrus and CA3 was preserved properly in both hemispheres. This allowed the identification and delimitation of each of the regions of analysis: the molecular, granule, and hilar dentate gyrus layers, as well as the oriens, pyramidal, lucidum, and RLM CA3 strata (Figures 3B,C). The anatomical boundaries and criteria to define each layer of the dentate gyrus and each CA3 strata were established according to Amaral and Witter (1989), Gulyás et al. (2001), Amaral et al. (2007), and Paxinos and Watson (2007). Thus, in the dentate gyrus (−1.72 to −6.84 mm posterior to Bregma), the granule layer is the compact layer that contains the somas of the granule cells. KCC2 staining was observed around the neuronal somas, i.e., in the plasma membrane, whereas the somas of the granule cells were not dyed (Figure 3E). Below this layer is the molecular layer containing mainly the dendrites of the granule cells in a dark tone. The strongest neuropil staining of the entire hippocampus was observed in this layer (Figure 3D). Above the granular layer lies the hilar layer, where the labeling was weaker and delimited by the upper and lower borders of the dentate gyrus. KCC2 staining was observed around polymorphic cells and in neural processes (Figure 3F).

In CA3 (−1.72 mm to −6.12 mm posterior to Bregma) (Figure 3G), the compact zone of the somas of the pyramidal cells forms the pyramidal stratum, and peripheral KCC2 staining was observed in the somas of these cells, i.e., in the plasma membrane. Below the pyramidal layer is the dark-toned oriens stratum. Diffuse immunostaining was strongest in stratum oriens, which is delimited by the white matter of the alveus. The basal dendrites of the pyramidal cells are located here. Above the pyramidal layer lies the acellular stratum lucidum, which is very clear. The region contains the axons of the granule cells and the proximal apical dendrites of the pyramidal cells. The only field that presents a stratum lucidum is the CA3 field; therefore, it delimits the CA3. Next are the radiata and lacunosum-moleculare strata. Because they are difficult to delimit, they were evaluated together, and contain the medial and distal apical dendrites of the pyramidal cells.

**Figure 4:**
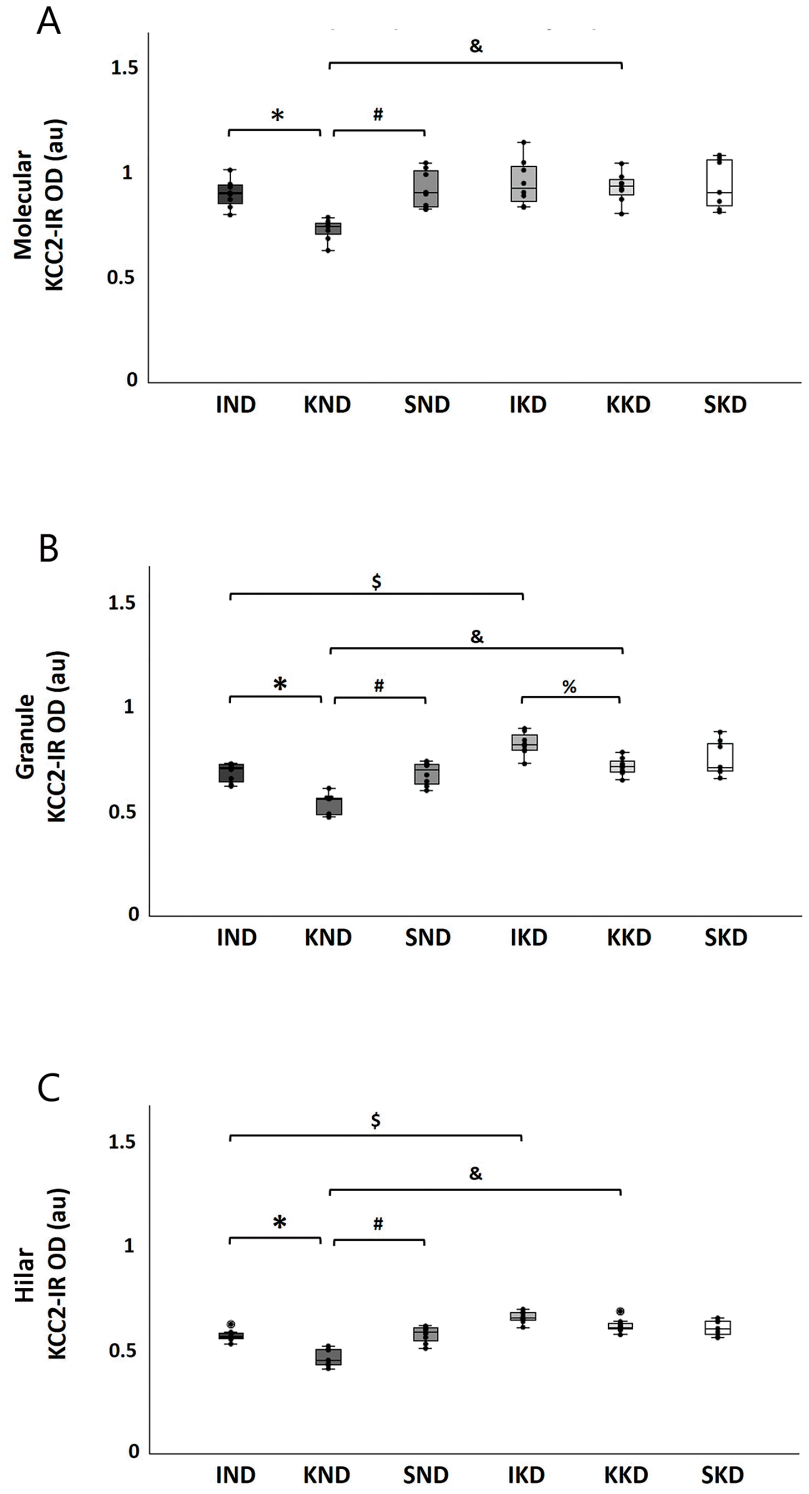
Boxplots with individual data points of KCC2 expression [KCC2-immunoreactivity optical density (KCC2-IR OD)] for each group in dentate gyrus layers: molecular **(A)**, granule **(B)**, and hilar **(C)**. The two-way ANOVA and Bonferroni’s *post hoc* test indicated that the ND-fed kindling group (KND) has a lower KCC2 expression when compared to the ND-fed intact group (IND) in molecular (**A**, *), granule (**B**, *), and hilar (**C**, *) layers and when compared to the ND-fed sham group (SND) in molecular (**A**, #), granule (**B**, #), and hilar (**C**, #) layers. However, these changes were not observed in the KD-fed groups (IKD, KKD, and SKD). Only in the granule layer had the KD-fed kindling group (KKD) lower KCC2 expression when compared with the KD-fed intact group (IKD) (**B**, %). The IKD group had more KCC2 expression than the IND group in granule (**B**, $) and hilar (**C**, $) layers. KKD has a higher KCC2 expression than the KND group in molecular (**A**, &), granule (**B**, &), and hilar (**C**, &) layers. In all comparisons, *p* < 0.001, except for 4B, % with *p* < 0.01. ND, normal diet; KD, ketogenic diet; SND, ND-fed sham.

Dentate gyrus: Two-way ANOVA showed that the Diet × Manipulation interaction was significant in the molecular layer (F(2, 40) = 4.503, *p* < 0.05, η^2^ = 0.184). The Bonferroni’s *post hoc* test indicated that when animals were fed with ND, the kindling group (KND) had presented significantly lower (18.62%) KCC2 expression than the intact group (IND) (*p* < 0.001) (Figure 4A, *) and sham group (SND) (20.30%; *p* < 0.001) (Figure 4A, #). On the contrary, when animals were fed with KD, the kindling group (KKD) was not significantly different from the intact group (IKD) and the sham group (SKD). In the kindling groups, the KD-fed group (KKD) exhibited higher KCC2 expression than the ND-fed group (KND) (26.8%; *p* < 0.001) (Figure 4A, &).

In the granule layer, the Diet × Manipulation interaction was significant (F(2, 40) = 3.325, *p* < 0.05, η^2^ = 0.143). In this case, the Bonferroni’s *post hoc* test evidenced that when animals were fed with ND, the kindling group (KND) presented significantly lower KCC2 expression than the intact group (IND) (22.15%; *p* < 0.001) (Figure 4B, *) and the sham group (SND) (21.47%; *p* < 0.001) (Figure 4B, #). When the animals were fed with KD, no significant difference was observed between the KKD and SKD groups. However, the KKD group had a significantly lower KCC2 expression than the IKD group (13.03%; *p* < 0.01) (Figure 4B, %), because IKD significantly increased the expression of KCC2, and this was even significantly higher than that in the IND group (19.67%; *p* < 0.001) (Figure 4B, $). The KKD group exhibited a significantly higher KCC2 expression than the KND group (33.70%; *p* < 0.001) (Figure 4B, &).

In the hilar layer, similar to the other layers of the dentate gyrus, the Diet × Manipulation interaction was significant (F(2, 40) =10.961, *p* < 0.001, η^2^ = 0.354). In cases that received ND, the Bonferroni’s *post hoc* test indicated that the kindling group (KND) had significantly lower KCC2 expression than the intact group (IND) (18.55%; *p* < 0.001) (Figure 4C, *) and the sham group (SND) (19.6%; *p* < 0.001) (Figure 4C, #). In contrast, when animals were fed with KD, no significant differences were observed in the IKD, KKD, and SKD groups. For the kindling groups, rats fed with a KD diet (KKD) had significantly higher KCC2 expression than those fed with a normal diet (KND) (33.18%; *p* < 0.001) (Figure 4C, &). A similar pattern was observed for the intact groups; the IKD group had significantly higher KCC2 expression than the IND group (15.01%; *p* < 0.001) (Figure 4C, $).

**Figure 5:**
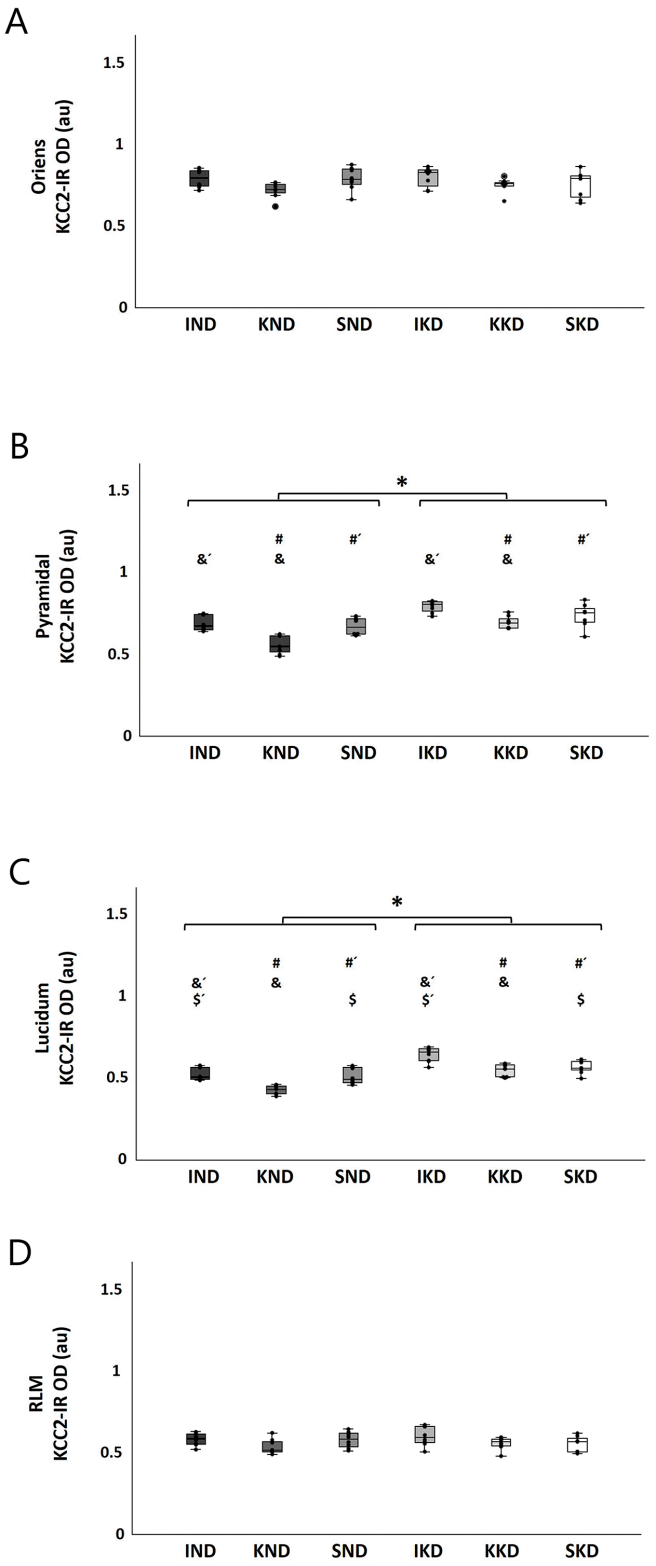
Boxplots with individual data points of KCC2 expression [KCC2-immunoreactivity optical density (KCC2-IR OD)] for each group in CA3 strata: oriens **(A)**, pyramidal **(B)**, lucidum **(C)**, and RLM **(D)**. The two-way ANOVA and Tukey’s *post hoc* test indicated that there were no significant changes in the oriens **(A)** and RLM strata **(D)**. The KD-fed groups (IKD, KKD, and SKD) had greater KCC2 expression than the ND-fed groups (IND, KND, and SND) in the pyramidal stratum (**B**, *) and the lucidum stratum (**C**, *) (*p* < 0.001). Kindling groups (KND and KKD) had a lower KCC2 expression than the intact groups (IND and IKD) in the pyramidal (**B**, &, &´) and lucidum (**C**, &, &´) strata (*p* < 0.001), and it was also lesser in relation to sham groups (SND and SKD) in pyramidal (**A**, #, #´) and lucidum (**B**, #, #´) strata (*p* < 0.01). A significant difference between sham groups (SND and SKD) and intact groups (IND and IKD) was observed only in the stratum lucidum (**C**, $, $´) (*p* < 0.01). ND, normal diet; KD, ketogenic diet; IND, ND-fed intact; KND, ND-fed with amygdala kindling; SND, ND-fed sham; IKD, KD-fed intact; KKD, KD-fed with amygdala kindling; SKD, KD-fed sham.

CA3: In the oriens stratum, two-way ANOVA did not reveal significant interactions or main effects on this layer (Figure 5A).

In contrast, in the pyramidal stratum, two-way ANOVA revealed a significant main effect of Diet (F(1, 40) = 44.58, *p* < 0.001, η^2^ = 0.527). Tukey’s *post hoc* test indicated that the KD-fed groups (IKD, KKD, and SKD) had significantly greater KCC2 expression than the ND-fed groups (IND, KND, and SND) (16.06%, *p* < 0.001) (Figure 5B, *). The factor Manipulation was also significant (F(2, 40) = 19.06, *p* < 0.001, η^2^ = 0.1488). The Tukey’s *post- hoc* test showed that the kindling groups (KND and KKD) had significantly lower KCC2 expression than the intact groups (IND and IKD) (16.71%, *p* < 0.001) (Figure 5B, &, &´) and the sham groups (SND and SKD) (12.95%; *p* < 0.01) (Figure 5B, #, #´).

In the lucidum stratum, two-way ANOVA showed a significant main effect of Diet (F(1, 40) = 66.475, *p* < 0.001, η^2^ = 0.624). The Tukey’s *post hoc* test found that the KD-fed groups (IKD, KKD, and SKD) had significantly greater KCC2 expression than the ND-fed groups (IND, KND, and SND) (20%; *p* < 0.001) (Figure 5C, *). For the Manipulation factor (F(2, 40) = 21.844, *p* < 0.001, η^2^ = 0.522), it was observed by means of Tukey’s *post hoc* test that the kindling groups (KND and KKD) had lower significant KCC2 expression than the intact groups (IND and IKD) (16.38%; *p* < 0.001) (Figure 4C, &, &´) and the sham groups (SND and SKD) (9.59%; *p* < 0.001) (Figure 5C, #, #´). A significant difference was observed between the sham groups (SND and SKD) and intact groups (IND and IKD), with this being higher in the intact groups (7.67%; *p* < 0.01) (Figure 5C, $, $´).

Finally, in the RLM stratum, two-way ANOVA did not reveal significant interactions or main effects on this layer (Figure 5D).

### 3.8 Pearson’s correlation coefficient between KCC2-IR levels and ADD

Pearson’s correlation test revealed significant negative correlations in all layers of the dentate gyrus: molecular r(45) = − 0.810, *p* < 0.001; granule r(45) = − 0.725, *p* < 0.001; and hilar r(45) = − 0.835, *p* < 0.001; and in the CA3 strata: pyramidal r(45) = − 0.719, *p* < 0.001; and lucidum r(45) = − 0.747, *p* < 0.001. These findings indicate that in these regions, the higher the KCC2 levels, the shorter the ADD in generalized seizures. The correlation in oriens and RLM strata was not significant.

## 4 Discussion

We demonstrated that KD has an antiepileptic function in amygdala kindling by reducing ADD in generalized seizures. In addition, the present study provides experimental evidence suggesting that KD has a putative neuroprotective effect by preventing the kindling-induced reduction of KCC2 expression in the molecular, granule, and hilar dentate gyrus layers and pyramidal and lucidum CA3 strata. The findings indicate a differential effect of KD on KCC2 expression by region and layer or stratum. The higher the KCC2 expression levels, the shorter the ADD.

Considering the importance of GABA-mediated neurotransmission, regulation of intracellular chloride concentration by cation-chloride cotransporter, specifically KCC2, in epilepsy and the beneficial effects of KD in this disease, the results presented in this study include data on body weight, glucose and β-hydroxybutyrate concentration, as well as ADD, latency and duration of stages in rats fed with a KD under the amygdala kindling model. In addition, we obtained detailed information on the regional expression of KCC2 in different layers of the dentate gyrus and CA3 strata.

In concordance with previous studies, we found that KD-fed rats experienced a significant decrease in glucose concentration (Jiang et al., 2016) and an increase in β-hydroxybutyrate levels (Gómez-Lira et al., 2011; Jiang et al., 2012, 2016; Granados-Rojas et al., 2020). β-hydroxybutyrate concentration was taken as a measure of ketonemia; thus, this result confirmed the efficacy of the diet used in this study to induce ketosis until the end of the experiment (Jiang et al., 2012, 2016; Granados-Rojas et al., 2020; Meeusen et al., 2024). In addition, a decrease in body weight was observed in rats subjected to KD and kindling (Jiang et al., 2012, 2016). This may herald the metabolic effects of KD on epilepsy.

In this study, the KD was started before the establishment of the amygdala kindling model, an experimental paradigm widely used to evaluate the protective effect of the KD. In line with the above, the evaluation of the protective effect of the KD by Jiang et al. (2012) was performed before starting amygdala kindling. In the same manner, Wang et al. (2016) and Jiang et al. (2016) performed the same evaluation even before pentylenetetrazole (PTZ) kindling. Moreover, other authors provided other types of diets, such as caloric restriction, before amygdala kindling (Phillips-Farfán et al., 2015; Rubio-Osornio et al., 2018). Nonetheless, we propose that future studies are needed to evaluate the impact of KD on KCC2 expression after establishing amygdala kindling. In the study by Hori et al. (1997) in which amygdala kindling was first established and then a KD was provided, no alterations were found in the kindling model, however, the diet used in this study contained zero carbohydrates.

The amygdala kindling model allows researchers to study the neurobiology of epilepsy in rats fed ND and KD diets (Hori et al., 1997; Hu et al., 2011; Jiang et al., 2012). Furthermore, the kindling model used in this study reflects the generalized tonic–clonic seizures that show a higher incidence in the epileptic population. Focusing on specific kindling outcomes, a significant reduction in ADD was observed in the KD-fed group compared with the ND-fed group (Jiang et al., 2012; Wang et al., 2016; Meeusen et al., 2024). This suggests that KD has a beneficial effect on epileptic activity (Campbell et al., 2015; Murugan and Boison, 2020; Meeusen et al., 2024). In terms of latency, a significant increase in the time taken to reach stage 3 was observed in the KD-fed group compared with the ND-fed group (Jiang et al., 2012; Wang et al., 2016). This may indicate a higher resistance to seizure induction, which could be a positive effect of KD (Qiao et al., 2024). When kindling stages 4 and 5 were combined to analyze the latency corresponding to the generalized seizure condition, an increase in latency was observed in KD-fed animals (Meeusen et al., 2024), indicating that more time is required to establish the generalized seizure condition in KD-fed animals, thus observing the beneficial effect of this diet in this epilepsy model. In the KD-fed group, rats stayed in stage 4 (generalized seizures) for a longer duration than the ND-fed group. The putative neuroprotective effect of KD in the amygdala kindling model was demonstrated by the increase in ADD as well as the latency and duration of generalized seizures. This result coincides with the data reported in other studies (Jiang et al., 2012, 2016; Wang et al., 2016).

The kindling model has been used to study the expression of cation-chloride cotransporter (Rivera et al., 2002; Okabe et al., 2003; Ding et al., 2013; Wang et al., 2016; Tescarollo et al., 2023). Regarding KCC2 expression, a differential effect of KD was observed by cerebral region, since the dentate gyrus was the brain region with the greatest positive changes when compared with CA3. The differential effect of KD was also observed by layer and stratum. The granular layer of the dentate gyrus exhibited the largest changes in KCC2 expression, whereas in CA3, changes were observed only in the pyramidal and lucidum strata. The oriens, radiate, and lacunosum-moleculare strata remained unchanged.

Previous studies have reported that hippocampal kindling reduces KCC2 expression levels in the hippocampus (Rivera et al., 2002), PTZ kindling in the cerebral cortex (Wang et al., 2016), as well as optogenetic kindling in the hippocampus (Tescarollo et al., 2023) in animals fed a normal diet. A similar situation was observed in the present study, where our ND-fed animals that were subjected to kindling showed a reduction of KCC2 expression in the molecular, granular, and hilar layers of the dentate gyrus and pyramidal and lucidum CA3 strata when compared with both intact and sham animals. However, when the KD-fed groups were analyzed, this reduction was not observed in the molecular, granular, and hilar layers of the dentate gyrus. These findings suggest a putative protective effect of KD by preventing the reduction of KCC2 expression induced by kindling in the dentate gyrus layers and cerebral cortex (Wang et al., 2016). Although a reduction in KCC2 expression was observed in the granular layer of KD-fed rats subjected to kindling when compared with the ND-fed rats with kindling, this effect is probably due to the significant increase presented by the latter group. In addition, the KCC2 expression levels of the KD-fed group subjected to kindling did not differ from the ND-fed intact and sham groups.

It must be noted that KD *per se* induces an increase in KCC2 expression levels in the granular and hilar dentate gyrus layers, supporting previous results (Granados-Rojas et al., 2020). KD *per se* also increases KCC2 expression level in the cerebral cortex (Wang et al., 2016). These results indicate that different brain regions increase KCC2 expression in response to KD *per se*.

When KCC2 expression levels were compared among the kindled groups, higher expression was observed in the KD-fed group than in the ND-fed group. This result coincides with that reported by Wang et al. (2016) in the cerebral cortex. In the CA3 region, it was found that kindling reduces KCC2 expression in the pyramidal and lucidum strata compared with the intact and sham groups (Rivera et al., 2002) in both diet groups. The CA3 was the region that exhibited the least changes, indicating that KD possibly exerts its protective action mainly through the dentate gyrus. The sham groups on both diets had less KCC2 expression than the intact groups, suggesting that surgical manipulation reduces KCC2, as was observed in trauma.

In the specific discussion of kindling, it may be noted that KD seems to modulate KCC2 expression differently in the dentate gyrus and CA3 during kindling. In the dentate gyrus, KD appears to preserve KCC2 expression, which could contribute to its anticonvulsant effects observed in kindling ADD and latency. In CA3, the overall decrement in KCC2 expression in the kindling groups on both diets suggests a common response to increased excitability, independent of diet. It is important to note that these results are specific to the kindling model used in this study and that the precise relationship between KD and KCC2 expression may vary in other epilepsy models.

The increase in KCC2 in the dentate gyrus of the KD-fed kindling group compared with the ND-fed kindling group was also reported in a PTZ kindling model (Wang et al., 2016). This finding explains the reduction in ADD during the generalized seizure phase as well as the need for more sessions to reach this phase. The negative correlation observed between KCC2 expression and ADD during generalized seizures suggests a protective effect of diet on KCC2 in the dentate gyrus and CA3 in epilepsy.

In the adult brain, granule cells in the dentate gyrus are strongly inhibited by multiple variants of interneurons. This condition endows the dentate gyrus with the properties of a tightly regulated filter, limiting throughput between the entorhinal cortex and the hippocampus (Pathak et al., 2007). In the dentate gyrus, the granular layer contains the somas of granule cells. The dendrites of these cells are located in the molecular layer that receives efferences from the entorhinal cortex, while the hilar layer contains the axons of granule cells and various types of interneurons. On the other hand, in the CA3 area, the somas of pyramidal cells are found in the pyramidal stratum, while the proximal apical dendrites of these neurons are located in the lucidum stratum, with this being the place where mossy fibers or axons of granule cells arrive to make synapses with thorny excrescence dendrites of pyramidal cells. The prevention of the reduction of KCC2 expression produced by kindling, coupled with the increase in the expression of the same in the molecular, granule and hilar layers of the dentate gyrus, as well as in the pyramidal and lucidum strata of the KD-fed animals subjected to stimulation of the amygdala, suggests that in these layers and strata are where the propagation of excessive or aberrant activity to the circuit is inhibited, which makes the hippocampus less prone to seizures (Bonislawski et al., 2007). Future studies are needed to support this idea. It seems that the main region with the greatest beneficial effects is the dentate gyrus, which is very important since this region is highly epileptogenic (Bonislawski et al., 2007) and is also recognized as the entrance to hippocampal formation. On the other hand, in CA3, which organizes the response that flows to CA1 (the main exit of the hippocampal formation), the strata with the greatest changes were the pyramidal and lucidum.

The preservation of KCC2 expression levels in the dentate gyrus and CA3 in KD-fed animals under amygdala kindling reported in the present study complements previous reports carried out in the cerebral cortex of rats fed with a KD under PTZ kindling (Wang et al., 2016). This indicates that this effect may be general in the brain. This supports the proposal that the beneficial effect of KD on kindling observed in the present and other studies could be due to the increase of KCC2 not only in the cortex but also in the dentate gyrus and CA3. The protective effect of KD has also been observed in preventing neuronal loss in the CA1 area of the hippocampus in amygdaloid kindling (Jiang et al., 2012) and in attenuating spatial and item memory impairment in PTZ-induced seizures (Jiang et al., 2016; Wang et al., 2021).

The KCC2 function is to extract intracellular Cl^−^ to maintain low levels of Cl^−^ in neurons (Duy et al., 2019). Low concentrations of this ion in neurons lead to hyperpolarizing currents regulated by the GABA_A_ receptor. This condition reduces epileptiform discharge or convulsive activity and promotes the inhibitory response of GABA by reducing neuronal excitability. Therefore, the increase and maintenance of KCC2 observed in animals fed with KD could be the mechanism, or one of the mechanisms, of this diet in epilepsy. However, future functional studies are necessary. The causal relationship between KCC2 modulation and the antiepileptic effects of KD should be further investigated.

Several studies have shown that a KD modifies synaptic function, reduces neuronal excitability, and decreases epileptic activity in the hippocampus of rodents. These effects are related to metabolic changes, such as increased ketone bodies, neurotransmitter regulation (GABA/glutamate), modulation of ion channels, and reduction of neuroinflammation. For example, a study by Stafstrom and Rho (2012) highlighted how the KD alters neuronal excitability and neurotransmitter balance and reduces seizure frequency in animal models. Another study by Lang et al. (2016) also demonstrated the role of ketone bodies in modulating synaptic function and enhancing inhibitory neurotransmission. Furthermore, Kasper (2020) found that a KD reduces epileptic activity by regulating ion channels and decreasing neuroinflammation in the hippocampus. It is well known that there are sex differences in epilepsy. Susceptibility to excitability episodes and the occurrence of epileptic seizures are higher in men than in women (Reddy et al., 2021). Among the proposed molecular mechanisms underlying sex differences in seizure susceptibility are steroid hormones and neuronal chloride homeostasis regulated by the cation-chloride cotransporter NKCC1 and KCC2 (Reddy et al., 2021). We used epileptic male rats because female rats experience significant hormonal fluctuations throughout their estrous cycle. These hormonal fluctuations may influence neuronal activity and seizure susceptibility. On the other hand, there is a sexually dimorphic expression of KCC2, which is higher in females than in males (Galanopoulou and Moshé, 2003; Galanopoulou, 2008). The results obtained in the present study contribute to a better understanding of the effect of KD on epilepsy control; however, it is important to note that observations of KCC2 expression in the dentate gyrus and CA3 might change in epileptic female rats. Future studies are needed to address this issue.

In conclusion, KD has an antiepileptic function in amygdala kindling by reducing ADD in generalized seizures. In addition, KD has a putative neuroprotective effect by preventing the kindling-induced reduction of KCC2 expression in the molecular, granule, and hilar dentate gyrus layers and pyramidal and lucidum CA3 strata. Increased KCC2 expression levels are related to a shorter duration of generalized seizures. These results could explain, at least in part, the beneficial effect of KD in epilepsy.

## Data Availability

The original contributions presented in the study are included in the article/supplementary material, further inquiries can be directed to the corresponding authors.

## References

[ref1] Amaral D. G. Scharfman H. E. Lavenex P. (2007). The dentate gyrus: fundamental neuroanatomical organization (dentate gyrus for dummies). Prog. Brain Res. 163, 3–22. doi: 10.1016/S0079-6123(07)63001-5 17765709 PMC2492885

[ref2] Amaral D. G. Witter M. P. (1989). The three-dimensional organization of the hippocampal formation: a review of anatomical data. Neuroscience 31, 571–591. doi: 10.1016/0306-4522(89)90424-7. PMID: 2687721, PMID: 2687721

[ref3] Aronica E. Boer K. Redeker S. Spliet W. G. M. van Rijen P. C. Troost D.. (2007). Differential expression patterns of chloride transporters, Na^+^-K^+^-2Cl^−^-cotransporter and K^+^-cl^−^-cotransporter, in epilepsy-associated malformations of cortical development. Neuroscience 145, 185–196. doi: 10.1016/j.neuroscience.2006.11.041, PMID: 17207578

[ref4] Belperio G. Corso C. Duarte C. B. Mele M. (2022). Molecular mechanisms of epilepsy: the role of the chloride transporter KCC2. J. Mol. Neurosci. 72, 1500–1515. doi: 10.1007/s12031-022-02041-7, PMID: 35819636

[ref5] Bonislawski D. P. Schwarzbach E. Cohen A. S. (2007). Brain injury impairs dentate gyrus inhibitory efficacy. Neurobiol. Dis. 25, 163–169. doi: 10.1016/j.nbd.2006.09.002, PMID: 17045484 PMC1713625

[ref6] Campbell S. L. Robel S. Cuddapah V. A. Robert S. Buckingham S. C. Kahle K. T.. (2015). GABAergic disinhibition and impaired KCC2 cotransporter activity underlie tumor-associated epilepsy. Glia 63, 23–36. doi: 10.1002/glia.22730, PMID: 25066727 PMC4237714

[ref7] Cellot G. Cherubini E. (2014). GABAergic signaling as therapeutic target for autism spectrum disorders. Front. Pediatr. 2:70. doi: 10.3389/fped.2014.00070, PMID: 25072038 PMC4085902

[ref8] Chen L. Wan L. Wu Z. Ren W. Huang Y. Qian B.. (2017). KCC2 downregulation facilitates epileptic seizures. Sci. Rep. 7:156. doi: 10.1038/s41598-017-00196-7, PMID: 28279020 PMC5427808

[ref9] Di Cristo G. Awad P. N. Hamidi S. Avoli M. (2018). KCC2, epileptiform synchronization, and epileptic disorders. Prog. Neurobiol. 162, 1–16. doi: 10.1016/j.pneurobio.2017.11.002, PMID: 29197650

[ref10] Ding Y. Wang S. Jiang Y. Yang Y. Zhang M. Guo Y.. (2013). Fructose-1, 6-diphosphate protects against epileptogenesis by modifying cation-chloride co-transporters in a model of amygdaloid-kindling temporal epilepticus. Brain Res. 1539, 87–94. doi: 10.1016/j.brainres.2013.09.042, PMID: 24095797

[ref11] Doyen M. Lambert C. Roeder E. Boutley H. Chen B. Pierson J.. (2024). Assessment of a one-week ketogenic diet on brain glycolytic metabolism and on the status epilepticus stage of a lithium-pilocarpine rat model. Sci. Rep. 14:5063. doi: 10.1038/s41598-024-53824-4, PMID: 38424459 PMC10904769

[ref12] Duy P. Q. David W. B. Kahle K. T. (2019). Identification of KCC2 mutations in human epilepsy suggests strategies for therapeutic transporter modulation. Front. Cell. Neurosci. 13:515. doi: 10.3389/fncel.2019.00515, PMID: 31803025 PMC6873151

[ref13] Dyńka D. Kowalcze K. Paziewska A. (2022). The role of ketogenic diet in the treatment of neurological diseases. Nutrients 14:5003. doi: 10.3390/nu14235003, PMID: 36501033 PMC9739023

[ref14] El-Shafie A. M. Bahbah W. A. Abd El Naby S. A. Omar Z. A. Basma E. M. Hegazy A. A. A.. (2023). Impact of two ketogenic diet types in refractory childhood epilepsy. Pediatr. Res. 94, 1978–1989. doi: 10.1038/s41390-023-02554-w, PMID: 36906721 PMC10007663

[ref15] Fedorovich S. V. Voronina P. P. Wassem T. V. (2018). Ketogenic diet versus ketoacidosis: what determines the influence of ketone bodies on neurons? Neural Regen Res. 13, 2060–2063. doi: 10.4103/1673-5374.241442, PMID: 30323121 PMC6199956

[ref16] Fisher R. S. Acevedo C. Arzimanoglou A. Bogacz A. Cross J. H. Elger C. E.. (2014). ILAE official report: a practical clinical definition of epilepsy. Epilepsia 55, 475–482. doi: 10.1111/epi.12550, PMID: 24730690

[ref17] Galanopoulou A. S. (2008). Sexually dimorphic expression of KCC2 and GABA function. Epilepsy Res. 80, 99–113. doi: 10.1016/j.eplepsyres.2008.04.013, PMID: 18524541 PMC2613346

[ref18] Galanopoulou A. S. Moshé S. L. (2003). Role of sex hormones in the sexually dimorphic expression of KCC2 in rat substantia Nigra. Exp. Neurol. 184, 1003–1009. doi: 10.1016/S0014-4886(03)00387-X, PMID: 14769394

[ref19] Gharaylou Z. Oghabian M. A. Azizi Z. Hadjighassem M. (2019). Brain microstructural abnormalities correlate with KCC2 downregulation in refractory epilepsy. Neuroreport 30, 409–414. doi: 10.1097/WNR.0000000000001216, PMID: 30817684

[ref20] Goddard G. V. McIntyre D. C. Leech C. K. (1969). A permanent change in brain function resulting from daily electrical stimulation. Exp. Neurol. 25, 295–330. doi: 10.1016/0014-4886(69)90128-9, PMID: 4981856

[ref21] Gómez-Lira G. Mendoza-Torreblanca J. G. Granados-Rojas L. (2011). Ketogenic diet does not change NKCC1 and KCC2 expression in rat hippocampus. Epilepsy Res. 96, 166–171. doi: 10.1016/j.eplepsyres.2011.05.017 21684720

[ref22] Granados-Rojas L. Jerónimo-Cruz K. Juárez-Zepeda T. E. Tapia-Rodríguez M. Tovar A. R. Rodríguez-Jurado R.. (2020). Ketogenic diet provided during three months increases KCC2 expression but not NKCC1 in the rat dentate gyrus. Front. Neurosci. 14:673. doi: 10.3389/fnins.2020.00673, PMID: 32733191 PMC7358437

[ref23] Gulyás A. I. Sík A. Payne J. A. Kaila K. Freund T. F. (2001). The KCl cotransporter, KCC2, is highly expressed in the vicinity of excitatory synapses in the rat hippocampus. Eur. J. Neurosci. 13, 2205–2217. doi: 10.1046/j.0953-816x.2001.01600.x, PMID: 11454023

[ref24] Hori A. Tandon P. Holmes G. L. Stafstrom C. E. (1997). Ketogenic diet: effects on expression of kindled seizures and behavior in adult rats. Epilepsia 38, 750–758. doi: 10.1111/j.1528-1157.1997.tb01461.x, PMID: 9579901

[ref25] Hu X. L. Cheng X. Fei J. Xiong Z. Q. (2011). Neuron-restrictive silencer factor is not required for the antiepileptic effect of the ketogenic diet. Epilepsia 52, 1609–1616. doi: 10.1111/j.1528-1167.2011.03171.x, PMID: 21762439

[ref26] Jiang Y. Lu Y. Jia M. Wang X. Zhang Z. Hou Q.. (2016). Ketogenic diet attenuates spatial and item memory impairment in pentylenetetrazol-kindled rats. Brain Res. 1646, 451–458. doi: 10.1016/j.brainres.2016.06.029, PMID: 27343950

[ref27] Jiang Y. Yang Y. Wang S. Ding Y. Guo Y. Zhang M. M.. (2012). Ketogenic diet protects against epileptogenesis as well as neuronal loss in amygdaloid-kindling seizures. Neurosci. Lett. 508, 22–26. doi: 10.1016/j.neulet.2011.12.002, PMID: 22178860

[ref28] Juárez-Zepeda E. Rubio C. Molina-Valdespino D. Marín-Castañeda L. A. Vanoye-Carlo A. Granados-Rojas L. (2024). “The ketogenic diet in neuropsychiatric disorders” in The ketogenic diet reexamined, myth vs. reality. eds. Granados-Rojas L. Rubio C. (New York: NOVA), 61–91. doi: 10.52305/KSTQ4025

[ref29] Kaila K. Price T. J. Payne J. A. Puskarjov M. Voipio J. (2014). Cation-chloride cotransporters in neuronal development, plasticity and disease. Nat. Rev. Neurosci. 15, 637–654. doi: 10.1038/nrn3819, PMID: 25234263 PMC4294553

[ref30] Karlócai M. R. Wittner L. Tóth K. Maglóczky Z. Katarova Z. Rásonyi G.. (2016). Enhanced expression of potassium-chloride cotransporter KCC2 in human temporal lobe epilepsy. Brain Struct. Funct. 221, 3601–3615. doi: 10.1007/s00429-015-1122-8, PMID: 26427846

[ref31] Kasper D. (2020). Modulation of ion channels and reduction of neuroinflammation by the ketogenic diet in epilepsy models. Neurosci. Lett. 731:135037. doi: 10.1016/j.neulet.2020.135037

[ref32] Koumangoye R. Bastarache L. Delpire E. (2021). NKCC1: newly found as a human disease-causing ion transporter. Function 2:zqaa028. doi: 10.1093/function/zqaa028, PMID: 33345190 PMC7727275

[ref33] Kwan P. Arzimanoglou A. Berg A. T. Brodie M. J. Allen Hauser W. Mathern G.. (2010). Definition of drug resistant epilepsy: consensus proposal by the ad hoc task force of the ILAE commission on therapeutic strategies. Epilepsia 51, 1069–1077. doi: 10.1111/j.1528-1167.2009.02397.x, PMID: 19889013

[ref34] Lam P. Newland J. Faull R. L. M. Kwakowsky A. (2023). Cation-chloride cotransporters KCC2 and NKCC1 as therapeutic targets in neurological and neuropsychiatric disorders. Molecules 28:1344. doi: 10.3390/molecules28031344, PMID: 36771011 PMC9920462

[ref35] Lang J. McCune S. K. Bhatia R. (2016). Effects of the ketogenic diet on synaptic function and neurotransmitter regulation. Epilepsy Res. 118, 22–28. doi: 10.1016/j.eplepsyres.2015.10.003,, PMID: 26590798 PMC4819482

[ref36] Lee H. H. Deeb T. Z. Walker J. A. Davies P. A. Moss S. J. (2011). NMDA receptor activity downregulates KCC2 resulting in depolarizing GABA_A_ receptor-mediated currents. Nat. Neurosci. 14, 736–743. doi: 10.1038/nn.2806, PMID: 21532577 PMC3102766

[ref37] Martin-McGill K. J. Bresnahan R. Levy R. G. Cooper P. N. (2026). Ketogenic diets for drug-resistant epilepsy. Cochrane Database Syst. Rev. 6:CD001903. doi: 10.1002/14651858.CD001903.pub5, PMID: 32588435 PMC7387249

[ref38] McMoneagle E. Zhou J. Zhang S. Huang W. Josiah S. S. Ding K.. (2024). Neuronal K^+^-Cl^−^ cotransporter KCC2 as a promising drug target for epilepsy treatment. Acta Pharmacol. Sin. 45, 1–22. doi: 10.1038/s41401-023-01149-9, PMID: 37704745 PMC10770335

[ref39] Meeusen H. Kalf R. S. Broekaart D. W. M. Silva J. P. Verkuyl J. M. van Helvoort A.. (2024). Effective reduction in seizure severity and prevention of a fatty liver by a novel low ratio ketogenic diet composition in the rapid kindling rat model of epileptogenesis. Exp. Neurol. 379:114861. doi: 10.1016/j.expneurol.2024.114861, PMID: 38876196

[ref40] Murakami M. Tognini P. (2022). Molecular mechanisms underlying the bioactive properties of a ketogenic diet. Nutrients 14:782. doi: 10.3390/nu14040782, PMID: 35215432 PMC8879219

[ref41] Murugan M. Boison D. (2020). Ketogenic diet, neuroprotection, and antiepileptogenesis. Epilepsy Res. 167:106444. doi: 10.1016/j.eplepsyres.2020.106444, PMID: 32854046 PMC7655615

[ref42] Official Mexican Norm. (1999). Technical specifications for the production, care and use of laboratory animals. [NOM-062-ZOO-1999]. Official Journal of the Federation, Mexico. Available online at: https://www.gob.mx/cms/uploads/attachment/file/203498/NOM-062-ZOO-1999_220801.pdf

[ref43] Okabe A. Yokokura M. Toyoda H. Shimizu-Okabe C. Ohno K. Sato K.. (2003). Changes in chloride homeostasis-regulating gene expressions in the rat hippocampus following amygdala kindling. Brain Res. 990, 221–226. doi: 10.1016/s0006-8993(03)03528-5, PMID: 14568348

[ref44] Pathak H. R. Weissinger F. Terunuma M. Carlson G. C. Hsu F. C. Moss S. J.. (2007). Disrupted dentate granule cell chloride regulation enhances synaptic excitability during development of temporal lobe epilepsy. J. Neurosci. 27, 14012–14022. doi: 10.1523/JNEUROSCI.4390-07.2007, PMID: 18094240 PMC2211568

[ref45] Paxinos G. Watson C. (2007). The rat brain in stereotaxic coordinates. London: Academic Press. 10.1016/0165-0270(80)90021-7 6110810

[ref46] Perucca E. White H. S. Bialer M. (2023). New GABA-targeting therapies for the treatment of seizures and epilepsy: II. Treatments in clinical development. CNS Drugs 37, 781–795. doi: 10.1007/s40263-023-01025-4, PMID: 37603261 PMC10501930

[ref47] Phillips-Farfán B. V. Rubio Osornio Mdel C. Custodio Ramírez V. Paz Tres C. Carvajal Aguilera K. G. (2015). Caloric restriction protects against electrical kindling of the amygdala by inhibiting the mTOR signaling pathway. Front. Cell. Neurosci. 9:90. doi: 10.3389/fncel.2015.00090, PMID: 25814935 PMC4356078

[ref48] Pressey J. C. de Saint-Rome M. Raveendran V. A. Woodin M. A. (2023). Chloride transporters controlling neuronal excitability. Physiol. Rev. 103, 1095–1135. doi: 10.1152/physrev.00025.2021, PMID: 36302178

[ref49] Qiao Q. Tian S. Zhang Y. Che L. Li Q. Qu Z.. (2024). A ketogenic diet may improve cognitive function in rats with temporal lobe epilepsy by regulating endoplasmic reticulum stress and synaptic plasticity. Mol. Neurobiol. 61, 2249–2264. doi: 10.1007/s12035-023-03659-3, PMID: 37870676

[ref50] Racine J. (1972). Modification of seizure activity by electrical stimulation: II. Motor seizure. Electroencephalogr. Clin. Neurophysiol. 32, 281–294. doi: 10.1016/0013-4694(72)90177-0, PMID: 4110397

[ref51] Rasband W. S. (2018). ImageJ, U. S. National Institutes of Health, Bethesda, Maryland, USA. Available online at: https://imagej.net/ij/

[ref52] Reddy D. S. Thompson W. Calderara G. (2021). Molecular mechanisms of sex differences in epilepsy and seizure susceptibility in chemical, genetic and acquired epileptogenesis. Neurosci. Lett. 750:135753. doi: 10.1016/j.neulet.2021.135753, PMID: 33610673 PMC7994197

[ref53] Rivera C. Li H. Thomas-Crusells J. Lahtinen H. Viitanen T. Nanobashvili A.. (2002). BDNF-induced TrkB activation down-regulates the K^+^-Cl^−^ cotransporter KCC2 and impairs neuronal Cl^−^ extrusion. J. Cell Biol. 159, 747–752. doi: 10.1083/jcb.200209011, PMID: 12473684 PMC2173387

[ref54] Rubio-Osornio M. D. C. Custodio-Ramírez V. Calderón-Gámez D. Paz-Tres C. Carvajal-Aguilera K. G. Phillips-Farfán B. V. (2018). Metformin plus caloric restriction show anti-epileptic effects mediated by mTOR pathway inhibition. Cell. Mol. Neurobiol. 38, 1425–1438. doi: 10.1007/s10571-018-0611-8, PMID: 30132243 PMC11481834

[ref55] Ryu B. Nagappan S. Santos-Valencia F. Lee P. Rodriguez E. Lackie M.. (2021). Chronic loss of inhibition in piriform cortex following brief, daily optogenetic stimulation. Cell Rep. 35:109001. doi: 10.1016/j.celrep.2021.109001, PMID: 33882304 PMC8102022

[ref56] Shi J. Xin H. Shao Y. Dai S. Tan N. Li Z.. (2023). CRISPR-based KCC2 upregulation attenuates drug-resistant seizure in mouse models of epilepsy. Ann. Neurol. 94, 91–105. doi: 10.1002/ana.26656, PMID: 37014252

[ref57] Shimizu-Okabe C. Tanaka M. Matsuda K. Mihara T. Okabe A. Sato K.. (2011). KCC2 was downregulated in small neurons localized in epileptogenic human focal cortical dysplasia. Epilepsy Res. 93, 177–184. doi: 10.1016/j.eplepsyres.2010.12.008, PMID: 21256718

[ref58] Stafstrom C. E. Rho J. M. (2012). The ketogenic diet as an treatment paradigm for diverse neurological disorders. Front. Pharmacol. 3:59. doi: 10.3389/fphar.2012.00059, PMID: 22509165 PMC3321471

[ref59] Taddei E. Rosiles A. Hernandez L. Luna R. Rubio C. (2022). Apoptosis in the dentate nucleus following kindling-induced seizures in rats. CNS Neurol. Disord. Drug Targets 21, 511–519. doi: 10.2174/1871527320666211201161800 34852754

[ref60] Tescarollo F. C. Valdivia D. Chen S. Sun H. (2023). Unilateral optogenetic kindling of hippocampus leads to more severe impairments of the inhibitory signaling in the contralateral hippocampus. Front. Mol. Neurosci. 16:1268311. doi: 10.3389/fnmol.2023.1268311 37942301 PMC10627882

[ref61] Verma J. P. (2015). Repeated measures design for empirical researchers. New Jersey: Wiley.

[ref62] Wan L. Chen L. Yu J. Wang G. Wu Z. Qian B.. (2020). Coordinated downregulation of KCC2 and GABA_A_ receptor contributes to inhibitory dysfunction during seizure induction. Biochem. Biophys. Res. Commun. 532, 489–495. doi: 10.1016/j.bbrc.2020.08.082 32892950

[ref63] Wan L. Ren L. Chen L. Wang G. Liu X. Wang B. H.. (2018). M-Calpain activation facilitates seizure induced KCC2 down regulation. Front. Mol. Neurosci. 11:287. doi: 10.3389/fnmol.2018.00287, PMID: 30186110 PMC6110871

[ref64] Wang S. Ding Y. Yan D. X. Rong L. Z. Hong S. C. Jin B.. (2016). Effectiveness of ketogenic diet in pentylenetetrazol-induced and kindling rats as well as its potential mechanisms. Neurosci. Lett. 614, 1–6. doi: 10.1016/j.neulet.2015.12.058, PMID: 26751594

[ref65] Wang X. Huang S. Liu Y. Li D. Dang Y. Yang L. (2021). Effects of ketogenic diet on cognitive function in pentylenetetrazol-kindled rats. Epilepsy Res. 170:106534. doi: 10.1016/j.eplepsyres.2020.106534, PMID: 33385944

[ref66] Wang Z. Sun M. Zhao X. Jiang C. Li Y. Wang C. (2017). Study of breath acetone in a rat mode of 126 rats with type 1 diabetes. J. Anal. Bioanal. Tech. 8:1. doi: 10.4172/2155-9872.1000344, PMID: 39887974

[ref67] Wheless J. W. (2008). History of the ketogenic diet. Epilepsia 49, 3–5. doi: 10.1111/j.1528-1167.2008.01821.x 19049574

[ref68] World Health Organization. (2024). Epilepsy. Available online at: https://www.who.int/health-topics/epilepsy#tab=tab_3 (Accessed March 20, 2024).

